# Fright but not fight‐or‐flight: Violent video games elevated stress markers, but did not impact muscle function, memory recall or food intake, in a randomized trial in healthy young men

**DOI:** 10.1002/ajpa.24559

**Published:** 2022-05-24

**Authors:** Jonathan C. K. Wells, Panos Michael, Mary S. Fewtrell, Mario Siervo, Mario Cortina‐Borja

**Affiliations:** ^1^ Population, Policy and Practice Research and Teaching Department UCL Great Ormond Street Institute of Child Health London UK; ^2^ School of Life Sciences, Division of Physiology, Pharmacology and Neuroscience University of Nottingham, Queen's Medical Centre Nottingham UK

**Keywords:** appetite, blood pressure, life history theory, stress, video games

## Abstract

**Objectives:**

Regular video game playing has been linked with obesity, but the underlying mechanisms remain unclear. Drawing on evolutionary life history theory, we hypothesized that playing violent video games, through activating the stress response, might increase the immediate demand for fuel by muscle and brain tissue, resulting in elevated appetite and food consumption.

**Methods:**

We randomized 71 young adult men to play video games, involving either violent content or nonviolent puzzle‐solving, for 1 h. Over this period, we measured stress markers (blood pressure [BP], heart rate, visual‐analogue scale [VAS] self‐ratings), muscle function (handgrip strength) and cognitive function (memory recall test). Appetite was assessed by VAS, and by food intake using a test‐meal after the intervention. Linear mixed‐effects models were fitted to assess group effects and group:time effects.

**Results:**

During the intervention, the violent video game group showed elevated systolic BP (∆ = 4.7 mm Hg, 95% CI 1.0, 8.4) and reported feeling more alert but less calm or happy. They showed no difference in grip strength or memory recall. They reported lower feelings of “fullness” but consumed similar food‐energy during the test‐meal.

**Conclusions:**

Although playing a video game with violent content elevated physiological and perceived stress markers compared with a nonviolent game, this was not associated with markers of altered fuel distribution toward two tissues (muscle and brain) that contribute to the “fight‐or‐flight” response. Rather than more energy being allocated to the brain overall, energy may have been reallocated within the brain. This may explain why there was no compensatory increase in energy intake in the violent video game group.

## Graphical abstract

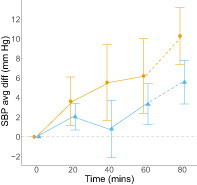

## 1 INTRODUCTION

Obesity is widely attributed to positive energy balance. This conceptual model, based on the first law of thermodynamics, states that energy cannot be created or destroyed, only transformed (Hall et al., 2012; Helmholtz, 1847). It seems purely a matter a logic that weight gain, generally involving fat accretion, is the inevitable product of energy intake exceeding energy expenditure. Much effort has therefore been directed to identifying potential causes of “excess energy intake,” or “insufficient energy expenditure” (Prentice & Jebb, 1995). According to this model, if daily energy intake were to systematically increase by a certain amount, weight should increase but eventually plateau, due to energy expenditure also rising in association with larger body mass. In practice, however, positive energy balance is often sustained over lengthy periods of time, indicated by chronic weight gain (Wells, 2013; Wells & Siervo, 2011). As an explanation for continued weight gain, however, the energy balance model itself is problematic, as it is simply a mathematical truism. Positive energy balance could be better regarded as a correlated symptom, rather than the cause, of weight gain (Taubes, 2008).

An alternative conceptual approach, attracting growing attention, attributes weight gain to regular or chronic perturbations of cellular metabolic regulation (Lustig, 2006, 2008; Taubes, 2008; Wells & Siervo, 2011). For example, diets inducing high insulin response promote lipogenesis, while also inducing lethargy and hunger even though the body has high circulating blood glucose levels (Lustig, 2006, 2008). In this approach, both positive energy balance and weight gain are considered consequences of perturbed metabolism, and the cause can be isolated in aspects of the individual's behavior, diet or environment that generate such metabolic perturbations. Among the potential candidates for perturbing metabolic regulation are sedentary behavior (Bergouignan et al., 2011; Duvivier et al., 2013; Healy, Dunstan, et al., 2008; Healy, Wijndaele, et al., 2008) and psychosocial stress (Chandola et al., 2006; Heraclides et al., 2009; Marniemi et al., 2002; Norberg et al., 2007). Importantly, these two factors may also interact with one another.

Under the energy balance paradigm, sedentary activities have been assumed to promote weight gain on account of their relatively low energy expenditure. However, alternative pathways could be involved, and one such pathway might involve alterations in appetite. For example, prolonged screen‐time has been associated in some studies not only with reduced physical activity but also with increased consumption of sweet and energy‐dense snacks (Cameron et al., 2016; Chaput, Klingenberg, et al., 2011; Marsh et al., 2013; Simons et al., 2015; Tabares‐Tabares et al., 2022; Thomson et al., 2008) and over the long term with higher BMI and obesity risk (Bennett et al., 2019; Fang et al., 2019; Hu et al., 2003; Liberali et al., 2021; Siervo et al., 2014).

In addition, some sedentary activities are characterized by elevated sensations of stress. For example, the best‐selling video games are “Action” or “Shooter” games incorporating high levels of violent content (Entertainment Software Association, 2014). Video game playing is already established to induce physiological and metabolic responses indicative of stress, such as a rise in sympathetic tone and cardiovascular load, accompanied by increases in heart rate and blood pressure (Anderson, 2004; Chaput, Visby, et al., 2011; Siervo et al., 2013; Siervo et al., 2018). An intriguing issue is whether the stress response to violent video games might modulate any association of sedentary behavior with appetite.

From an evolutionary perspective, there are several reasons for expecting inter‐relationships between the stress response, appetite and fuel metabolism. For many species of animal, resolving hunger often requires both physical exertion and dealing with physical threat (Watve, 2013). Carnivores require strength, speed, agility, and aggression when attacking prey animals. Even among non‐carnivores, subordinate individuals in social groups may have to grab food quickly while fending off aggressive dominant individuals (Watve, 2013). In such contexts, the stress response might be considered an adaptive mechanism when foraging requires dealing with physical threats, for example by diverting fuel to key traits such as muscle function and cognitive function. This perspective is consistent with evolutionary life‐history theory, which assumes that energy must be allocated in competition between competing traits and functions on a moment‐by‐moment basis, in order to optimize fitness (Hill, 1993). However, whether activating the stress response during sedentary behavior impacts the distribution of fuel to competing tissues has received little attention.

Two previous experiments randomized young adult men to one of three groups: violent video game, nonviolent video game, or television (TV) viewing. The first experiment, on normal‐weight men (BMI 18–25 kg/m^2^; *n* = 16 per group), demonstrated higher cardiovascular load (increased diastolic blood pressure) and decreased sensations of fullness in those playing the violent game for 1 h compared with the other two groups (Siervo et al., 2013). In the second experiment, on overweight or obese men (BMI >25 kg/m^2^; *n* = 24 per group), elevated stress markers were apparent in all those playing video games compared to those watching TV, but those playing the violent video game ate significantly more food than the controls in a post‐experiment test‐meal, whereas those playing the nonviolent video game showed no increase in consumption relative to the TV controls (Siervo et al., 2018). Another study randomized 94 young adults in groups to watch an action movie with or without sound, or an interview program. Those watching the action movie consumed significantly more food than those watching the interview, even when the movie was played silently (Tal et al., 2014). All of these studies indicate that exposure to violent media activates the stress response, and may propagate its effects to appetite and food intake.

One possibility is that the physiological and cognitive effects of such exposure to violent media might arise through the differential allocation of fuel (glucose) between competing organs/tissues. Under conditions of mild stress, plasma cortisol increases, resulting in the mobilization of glucose from liver stores, and hence an increase in circulating glucose (Chaput, Visby, et al., 2011), which may then be allocated differentially across tissues. The “Cerebral Supply Chain” model of Peters and colleagues hypothesizes that energy is allocated hierarchically within the body, with peripheral organs lower in the hierarchy, and the highest‐priority organ comprising the brain (Peters, 2011; Peters et al., 2011). At rest, the brain uses nearly two thirds of the body's circulating glucose, with glucose utilization rising by 12% under mild mental stress (Peters, 2011; Peters et al., 2011).

Catecholamine hormones secreted in response to stress impact a variety of physiological parameters, for example increasing cardiac output, blood pressure and triglyceride levels, and promoting contraction of the skeletal muscles, while reducing blood flow to the skin, kidneys, and gastrointestinal system (Cohen, 2009; Everly & Sobelman, 1987). Therefore, it is possible that both muscle and brain fuel demand might increase under stressful conditions, potentially thereby generating a mechanistic link between stress and appetite. Of relevance here, a recent study demonstrated competition for fuel between skeletal muscle tissue and the brain under an intense exercise load, with the brain appearing to take priority over muscle (Longman et al., 2017), though this study did not directly address the role of the stress response. Another study showed that exposure to a visual stimulus of aggressive behavior increased both cortisol and testosterone levels in highly trained male athletes (Cook & Crewther, 2012).

**FIGURE 1:**
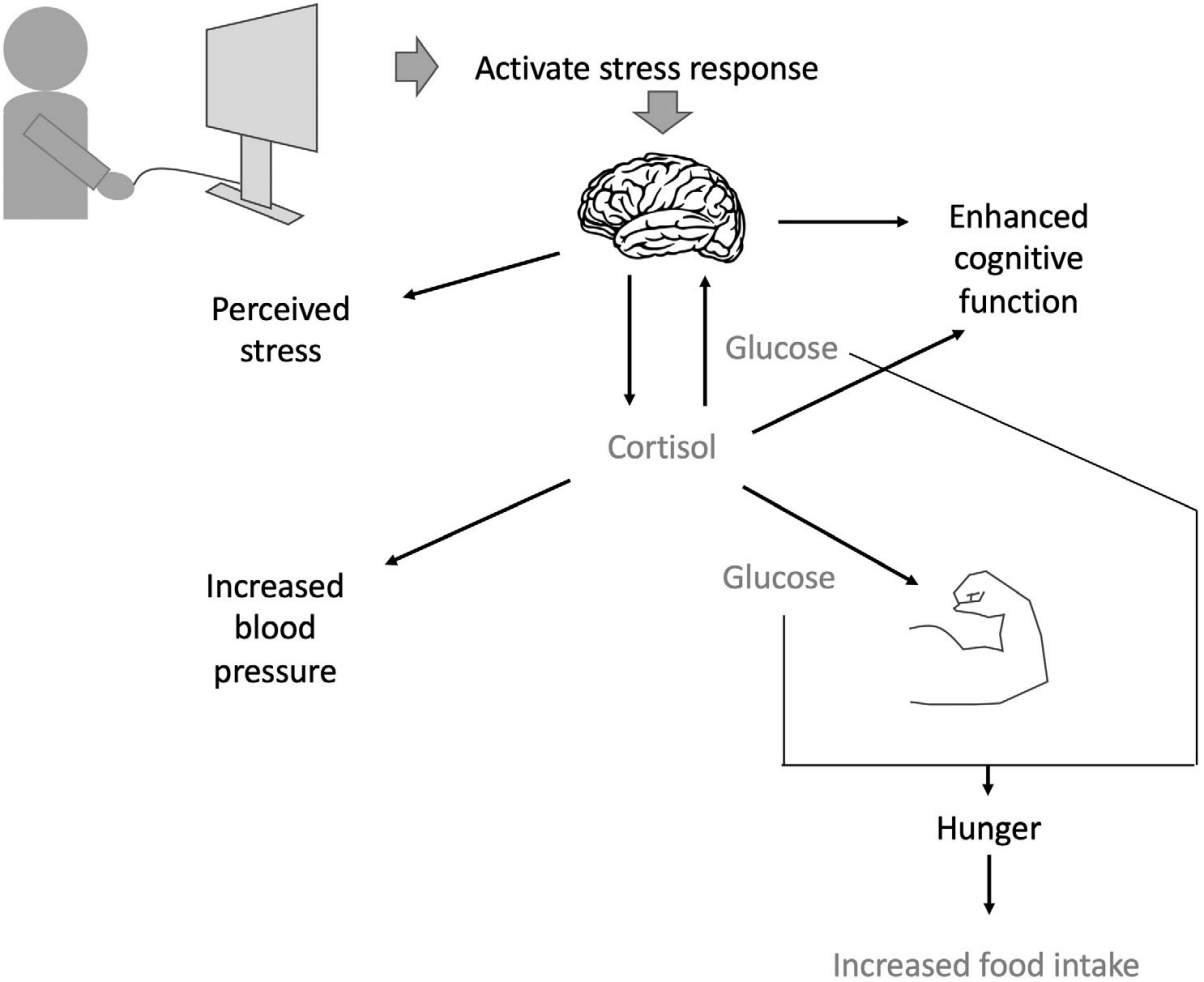
Schematic diagram illustrating the responses we hypothesized would occur following exposure to a violent video game, compared with playing a nonviolent game. We expected the stimulus of violent game participation to activate the stress response, leading to hormonal signals such as increased cortisol levels. We hypothesized that this would lead to increased physiological markers of stress (blood pressure) as well as perceived feelings of stress, along with greater allocation of fuel to muscle and cognitive function, indicated by performance on grip strength and memory recall tasks. Finally, expecting such increased fuel allocation to muscle and brain, we hypothesized increased feelings of hunger, and greater food intake at a test meal.

Here, we sought to test experimentally whether playing a violent video game would induce changes in markers of fuel supply to two tissues that contribute to the fight‐or‐flight response, muscle and brain, in comparison to playing a nonviolent puzzle‐solving video game. Both games involved solving tasks that differed markedly in the exposure to violent content. We hypothesized that playing the violent game would activate the stress response, and increase relative fuel allocation to muscle and brain, and that this would further impact appetite, assessed through a post‐game test‐meal. Our conceptual approach is illustrated in Figure 1.

To test our hypotheses, we conducted a randomized trial in healthy young adult men with body mass index (BMI) within the normal range. We restricted the trial to one sex, as the two sexes have different hormonal responses to activation of the stress response (Stephens et al., 2016). We selected young men, as playing video games is popular in this group (Siervo et al., 2014) and our findings might have public health significance. The primary outcome was total energy intake during the test‐meal, while we measured several physiological markers of the stress response, handgrip strength as a marker of muscle function, and memory recall as a marker of brain function.

## 2 METHODS

A two‐arm, parallel, randomized control experiment was carried out at the Childhood Nutrition Research Centre, University College London (Clinical-Trials.gov NCT02075827). Participants were informed that “the aim of this study is to establish the metabolic response to playing video games,” but were blinded to more specific hypotheses, in order to minimize any influence of individual expectations. The study was approved by the UCL Research Ethics Committee. All data were collected between February and May 2014.

### 2.1 Recruitment

The study was advertised using mass university emails, electronic bulletins, notice boards, flyers, social media, and word of mouth. The target was primarily university students as they are reported to engage in video gaming frequently, often for extended periods (Siervo et al., 2014; Wack & Tantleff‐Dunn, 2009). Prospective participants were sent an information sheet, and if interested arranged a telephone interview with author PM to confirm eligibility. Inclusion criteria were males aged 18–30 years, with BMI ≤25 kg/m^2^ and stable body weight (<3 kg change in the previous 3 months). Exclusion criteria were current smoking, alcohol consumption ≥21 units/week, medication that might interfere with the study outcomes, and comorbid psychiatric or physical illness such as depression, schizophrenia, uncontrolled hypertension, coronary heart disease, arteriopathy, and renal or liver dysfunction. Potential participants also needed to already be familiar with both games used in the study, “Call of Duty: Black Ops II” (COD; Activision Inc.) and “Little Big Planet 2” (LBP; Sony Computer Entertainment America LLC).

One participant per day was subsequently invited to attend the study from 09:30 to 11:30 a.m.; all participants were asked to fast overnight from 9 p.m., and to avoid caffeinated drinks for at least 12 h before arrival. Participants were compensated with £10 cash, and travel costs were reimbursed. On arrival, participants were asked to provide written informed consent, and to complete a “Participant Details” form to confirm their eligibility and fasting status.

### 2.2 Study protocol

The study was conducted in a standardized room, by author PM. On arrival, essential demographic data were obtained by questionnaire, and a set of baseline measurements was obtained. The protocols for all measurements are given in detail below. Anthropometry was performed, and baseline measurements were collected for heart rate, blood pressure (BP), handgrip strength and memory recall. In addition, assessments of emotional state and appetite using a visual analogue scale (VAS) were conducted. Once baseline assessments were complete, the participants were offered a standard breakfast consisting of a chocolate chip muffin (272 kcal, 12.8 g fat, 34.6 g carbohydrates, 3.8 g protein) and a bottle of water, representing approximately 10.8% of the estimated daily energy requirement. All participants consumed the breakfast within 10 minutes.

The randomization status was then determined by opening the envelope, following which the relevant video game was initiated. The game was interrupted for 3 minutes at 20 and 40 minutes for BP (measured in duplicate) and VAS measurements. At 60 minutes, the game was terminated and BP and VAS measurements repeated, followed by further tests of memory recall and handgrip strength. Heart rate was continuously monitored throughout the intervention. After the intervention, subjects were left alone to rest for 20 min. They were provided with ad libitum access to a variety of sweet, salty, savory and fatty snacks, as well as healthier options. At the end of the 20‐min period, BP and VAS outcomes were measured again, and the heart rate monitoring was ended.

### 2.3 Physical measurements

Anthropometric measurements (height, weight, waist circumference) were recorded in duplicate, and BMI (weight/height^2^) calculated. Although the protocol excluded overweight/obese individuals, in practice a cut‐off of ≤25.5 kg/m^2^was used when actually measuring participants, to allow for daily variation in body weight and clothing.

Heart rate was monitored throughout the study duration using a chest belt monitor (Polar H3 heart rate sensor) and a watch (Polar RS800CX Training Computer) worn on the right wrist. The belt detected heart rate every second and communicated the information wirelessly to the watch for data recording. At completion, the data were uploaded to a computer for analysis. The software calculated cumulative energy expenditure from the continuous heart rate data using manufacturers' prediction equations (Polar, 2014). Participants' age, height and weight were entered into the monitor for these calculations.

Participants were asked to sit still and silent for 3 min, resting their left arm on a table, feet flat on the floor. Systolic (SBP) and diastolic (DBP) blood pressure were recorded in triplicate (at baseline and end of study), or in duplicate (during the intervention), using a Datascope monitor (Accutorr Plus), with a 1‐min gap between readings. Mean values for SDP and DBP were used for analysis. Analysis of these data was restricted post hoc to a subsample, as the first 31 participants were measured with an arm‐cuff subsequently detected to be faulty. BP was measured at intervals through the intervention, as discussed in more detail below.

Handgrip strength was measured as a marker of muscle function using a dynamometer (Grip‐D, Takei, Japan). Participants stood with their shoulders adducted and their elbows held at 180°, and squeezed the dynamometer with maximum strength, starting with the right hand and alternating between the hands until a total of six readings were taken. The average score was recorded in kilograms (kg) to one decimal place. Grip strength was measured pre‐ and post‐intervention, and we assumed that any increase in grip strength over the course of the intervention would indicate increased resource allocation to muscle function.

### 2.4 Perceived sensations and cognitive measures

A visual analogue scale (VAS) was designed to quantitatively assess participants' feelings in response to various statements relating to their current emotional state and appetite. The scale comprises 18 statements each incorporating a 100 mm horizontal line marked to gauge responses ranging from “Very little” to “Very much.” Participants marked the line to reflect the extent to which they agreed with each statement, and digital calipers (Mitutoyo CD‐6″CP) were used to quantify the answers. The statements analyzed here were: “How stressed [or alert/calm/happy/full] do you feel?” The VAS was completed at intervals through the intervention, as discussed in more detail below.

The memory recall test comprised of a sheet displaying 30 colored and named objects. Participants were asked to look at the sheet for 30 s and memorize as many objects as possible. They were then allowed 1 min to write down a list of those recalled. All correctly recalled objects (identical wording to that on the list) were counted. Any incorrectly recalled objects, including similar objects or objects not displayed, were ignored. The memory test was carried out pre‐ and post‐intervention, using a different sheet with different items each time, and we assumed that any increase in the memory score over the course of the intervention would indicate increased resource allocation to cognitive function.

### 2.5 Randomization

Randomization to the two groups was carried out using a computerized scheme with a 1:1 allocation. The randomized numbers were placed in sealed envelopes numbered 1 to 76 (four extra envelopes were prepared, to allow for potential dropouts). The envelopes were prepared prior to recruitment by a third party unconnected with any other aspect of the study. Participants were allocated an ID number when recruited, and were asked to open a number‐matched envelope after the baseline measurements had been collected, to find out which game they would play.

### 2.6 Video games

COD is a first person shooter game with a “mature” Entertainment Software Rating Board (ESRB) score, while LBP is a platform game rated for “everyone.” They were selected because of being popular and easy to play, meaning that a large proportion of the video‐gaming public would be familiar with them (61). The game stages to be played were standardized and carefully selected to encompass either an element of continuous violence (COD) or continuous “pro‐social” puzzle‐solving activities (LBP). A Sony PlayStation 3 Dualshock button‐based controller and a 22‐inch, high‐definition television (Technika 22‐700) were used. The color contrast, brightness, and volume of the television were standardized for all participants. The video game intervention lasted for 1 h during which participants sat on a comfortable chair and were left alone in the room in between assessments, to avoid intrusion and to encourage engagement in the game.

### 2.7 Test meal

Snacks included chocolate bars, chocolate biscuits, cashew nuts, salted crisps, bananas, apples, orange juice. and a sugar‐sweetened soft drink (Sprite). They were also provided with a variety of magazines to read, and left alone to minimize any social effects on eating. The total consumption of snacks and drinks consumed during the rest period was recorded by counting the packages/bottles/cans opened; partially consumed content was counted as whole. The nutrient content of the individual items was obtained from the manufacturer's information, and the overall nutrient intake calculated. At the end of the resting period, the heart rate monitor was stopped.

### 2.8 Sample size calculation and statistics

Building on previous work, we aimed to detect a difference of 0.67 *SD* scores in our primary outcome, energy intake during the test‐meal. In our previous study, the *SD* of energy intake during the test meal was 339 kcal (Siervo et al., 2018), hence we aimed to detect a difference of ≤227 kcal. To detect this difference with 80% power at 5% significance, a sample size of 36 per group was required.

The distribution of the data was checked for normality using the Shapiro–Wilk test. Levene's test was used to assess equality of variance. Differences between the two groups were assessed using independent *t* tests. To aid in interpretation, differences between groups for VAS outcomes were expressed in terms of internal *z*‐scores, based on baseline data for the two groups combined. For variables with repeated measures (e.g., heart rate, BP, VAS) linear mixed‐effects models were fitted to assess group effects and group:time effects. To assess differences in the likelihood of consuming fruit or drinks, we calculated odds ratios for the COD group. We focus our discussion on the magnitude of effect, providing coefficients and 95% confidence intervals for all statistical tests. All calculations were performed in R version 4.0.5 (R Core Team, 2021).

## 3 RESULTS

Of 127 individuals expressing interest in the study, 19 did not respond for the phone interview. Of 108 screened, 31 did not satisfy the eligibility criteria, hence 77 were recruited. However, on arrival and further screening, 6 proved ineligible to participate, leaving 71 randomized successfully and who completed the protocol (Figure S1). There were no dropouts among participants, but as we had made provision for them, and generated IDs for 76 participants, the study ended with 35 in the COD group and 36 in the LBP group. There were no harms or unintended effects resulting from the intervention.

All participants had normal or corrected‐to‐normal vision. All had received tertiary education. Overall, 30% of the sample played video games daily, 48% weekly, and 22% monthly or less often.

Baseline characteristics are given in Table 1. Mean (*SD*) height was 177.2 (5.4) cm, and BMI was 22.3 (1.8) kg/m^2^, range 18.2 to 27.3 kg/m^2^. There was no difference between the two groups in age or any anthropometric measurements. The nonviolent group spent more time playing video games on a weekly basis.

**TABLE 1:** Description of the sample according to the type of video game played

	Violent game (*n* = 35)		Nonviolent game (*n* = 36)		Difference^a^
Baseline trait	Mean	*SD*	Mean	*SD*	Mean (95% CI)
Age (y)	21.7	2.7	22.6	3.2	−0.9 (−2.3, 0.5)
Height (cm)	177.3	4.2	177.1	6.5	0.3 (−2.3, 2.9)
Weight (kg)	69.9	6.7	70.2	6.3	−0.3 (−3.3, 2.8)
Waist girth (cm)	79.8	4.6	80.5	5.1	−0.7 (−3.1, 1.6)
BMI (m/kg^2^)	22.2	1.8	22.4	1.8	−0.2 (0.4, 0.7)
Video game time (h/week)	4.5	4.4	6.5	5.9	−2.0 (−4.5, 0.5)

^a^ Video game groups compared by independent samples *t* test.

### 3.1 Stress markers

**FIGURE 2:**
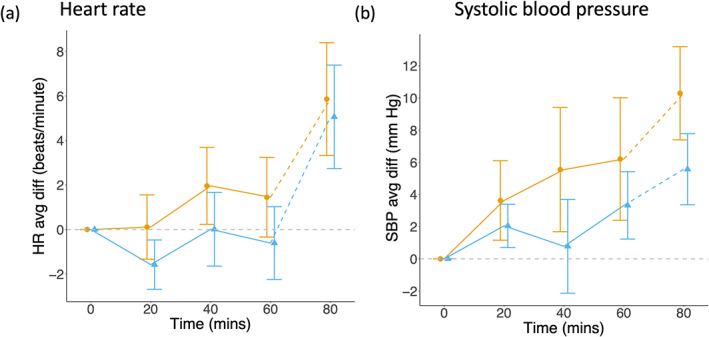
Mean change from baseline (delta) in stress markers. Data are mean and 95% CI. (a) The violent game group had consistently higher heart rate during the intervention by ~2 beats/min (95% CI −3, 6). (b) The violent game group demonstrated an increase in SBP during the game intervention, while the nonviolent group maintained a more or less constant SBP. This difference persisted after the intervention, and at the end of the test‐meal the difference between the groups was 4.7 mm Hg (95% CI 1.0, 8.4)

There was no difference between the groups in baseline heart rate (COD: mean 74.3, *SD* 10.6 beats/minute; LBP: mean 74.6, *SD* 11.4 beats/min). After the intervention began, the LBP group experienced a slight decrease and the COD group a slight increase in heart rate, such that mean of the COD group was ~2 beats/minute higher (95% CI −3, 6) during the intervention, equivalent to 0.1 z‐scores of the sample at baseline (Figure 2a).

In the subsample with BP data (*n* = 41), there was a marked increase in SBP during the intervention in the COD group, compared to the LBP group (Figure 2b). This contrast persisted at the end of the study, with a mean group difference of 4.7 (95% CI 1.0, 8.4) mm Hg, equivalent to 0.50 z‐scores of the sample at baseline. The equivalent increase in difference in DBP was smaller (∆ = 2.4 mm Hg, 95% CI −0.6, 5.3), equivalent to 0.34 z‐scores of the sample at baseline.

**FIGURE 3:**
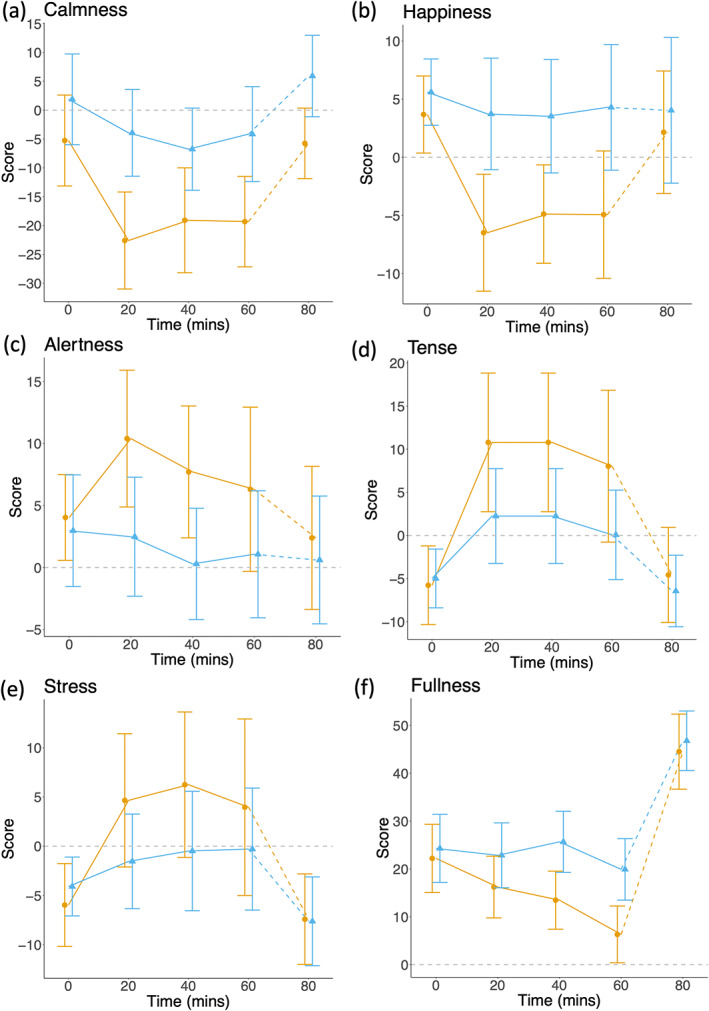
Mean change from baseline (delta) values for markers of feelings measured by the VAS (mm). Plotted data are mean and 95% CI, group differences are expressed in z‐scores relative to baseline data in the whole sample. (a) The violent video game group felt less calm both during (∆ = −0.86 *z*‐scores) and following the intervention (∆ = −0.69 *z*‐scores). (b) The violent video game group felt less happy (∆ = −0.64 *z*‐scores) during the intervention but not after the test‐meal. (c) The violent video game group felt more alert (∆ = 0.42 *z*‐scores) during the intervention but not after the test‐meal. (d) The violent video game group felt more tense (∆ = 0.62 *z*‐scores) during the intervention. (e) The violent video game group felt more stressed (∆ = 0.41 *z*‐scores) during the intervention. (f) The violent video game group showed a greater decline in feeling full during the intervention, but then demonstrated a greater increase in feeling full during the test meal period, so that by the end of the test‐meal they did not differ from the nonviolent group

By VAS, the COD group had reduced feelings of calmness both throughout (−18.9, 95% CI −32.4, −5.3) and following (∆ = −15.2, 95% CI −25.6, −4.7) the intervention (Figure 3a), equivalent to differences of −0.86 and − 0.69 *z*‐scores respectively. The COD group also had reduced feelings of happiness (∆ = −10.3, 95% CI −18.6, −2.0) (Figure 3b) and increased feelings of alertness (∆ = 9.5, 95% CI 0.7, 18.4) (Figure 3c) during the intervention, equivalent to −0.64 *z*‐scores and 0.42 *z*‐scores respectively. The COD group reported feeling more stressed (∆ = 8.9, 95% CI ‐1.4, 19.2) and more tense (∆ = 7.7, 95% CI −2.9, 18.3) during the intervention, equivalent to 0.62 *z*‐scores and 0.41 *z*‐scores respectively (Figure 3d,e).

### 3.2 Estimated energy expenditure

Mean cumulative energy expenditure calculated from the heart rate data did not differ significantly between the groups (COD Mean: 281.5 kcal, *SD* 18.3; LBP Mean: 271.0, *SD* 67.7; ∆ = 10.5, 95% CI −31.9, 52.9).

### 3.3 Markers of fuel distribution

There was no difference in handgrip strength at baseline, endline or in their change between these time‐points (Table 2). There was no significant difference in memory recall ability at baseline (∆ = 0.50,95% CI −0.54, 1.55), however at endline the score was higher in the COD group (∆ = 0.91, 95% CI −0.00, 1.82). Overall, these data indicated that the change during the intervention was slightly greater in the COD group (∆ = 0.40,95% CI −0.66, 1.46 (Table 2).

**TABLE 2:** Baseline and endline grip strength and cognitive function scores, and their changes over the 1 h intervention

	Violent game (*n* = 35)		Nonviolent game (*n* = 35)		Difference^a^	*SD* Difference^b^
Outcome	Mean	SD	Mean	SD	Mean (95% CI)	Mean
Grip strength						
Baseline score (kg)	37.1	6.9	37.8	6.6	−0.7 (−3.9, 2.6)	0.1
Endline score (kg)	37.1	6.6	37.5	6.6	−0.3 (−3.5, 2.8)	<0.1
Change in score (kg)	0.01	3.3	−0.3	3.6	0.3 (−1.3, 2.0)	<0.1
Memory recall test						
Baseline score (items)	10.2	2.0	9.7	2.4	0.5 (0.5, 1.6)	0.2
Endline score (items)	9.7	2.0	8.8	1.8	0.9 (−0.0, 1.8)	0.4
Change in score (items)	−0.5	2.4	−0.9	2.0	0.4 (−0.6, 1.5)	0.2

*Note*: Video game groups compared by independent samples *t* test.

^a^ Absolute difference, violent group–nonviolent group values.

^b^ Difference expressed in terms of z‐score of whole sample.

### 3.4 Appetite and food intake

By VAS, the COD group experienced a decline in the sensation of fullness compared with the nonviolent group over the 60‐min intervention (group by time ∆′ = −0.17, 95% CI −0.32, −0.03) (Figure 3f). During the post‐game “ad libitum snack” resting period, both groups showed an increase in the sensation of fullness and reached similar final levels. The gradient of this increase was greater in the COD group relative to the nonviolent group (group by time ∆′ = 0.57, 95% CI 0.06, 1.07).

The COD group consumed higher amounts of total energy (∆ = 26 kcal, 95% CI ‐132, 184), carbohydrate and sugar (Table 3), compared to the nonviolent game group, but these differences were all very small, and amounted to ~0.1 *SD* of the average intake. There was also no difference in the likelihood of the COD group consuming fruit (OR 0.81, 95% CI 0.45, 1.46), or an energy‐containing drink (OR 1.31, 95% CI 0.72, 2.38).

**TABLE 3:** Ad libitum food intake during the post‐intervention test‐meal

	Violent game (*n* = 35)		Nonviolent game (*n* = 36)		Difference^a^	*SD* difference^b^
Food intake	Mean	*SD*	Mean	*SD*	Mean (95% CI)	Mean
Energy (kcal)	810	331	784	335	26 (−132, 184)	0.1
Protein (g)	16.4	8.0	15.3	7.6	1.1 (−2.6, 4.8)	0.1
Carbohydrate (g)	94.3	36.8	91.6	33.1	2.7 (−13.8, 19.2)	0.1
Sugar (g)	68.2	27.4	63.6	22.7	4.6 (−7.3, 16.6)	0.2
Fat (g)	38.9	19.3	37.8	21.1	1.1 (−8.5, 10.7)	0.1
Salt (g)	0.58	0.44	0.65	0.43	−0.06 (−0.27, 0.14)	0.1

*Note*: Video game groups compared by independent samples *t* test.

^a^ Absolute difference, violent group–nonviolent group values.

^b^ Difference expressed in terms of ‐*score* of whole sample.

## 4 DISCUSSION

This study tested whether experimental activation of the stress response over 1 h would stimulate fuel demand in two tissues associated with the flight‐or‐fight response (muscle and brain), and whether this would result in increased appetite and greater food consumption immediately after the experiment. The study drew on an evolutionary life history framework, which assumes that energy is allocated differentially across competing organs and tissues in ways that maximize fitness. The findings could potentially help understand why regular video gaming has been associated with the risk of obesity (Siervo et al., 2014). Previously, we found that normal weight young men felt less full after playing a violent video game (Siervo et al., 2013), and that overweight young men playing a violent video game consumed more energy after the game had ended, compared to those watching TV, whereas those playing a nonviolent game showed no such increase in energy intake (Siervo et al., 2018).

In the present study, on men with BMI within the normal range, we found that compared to playing a nonviolent puzzle‐solving video game, the violent video game elevated several markers of stress (SBP and perceived feelings of alertness, accompanied by lower perceived feelings of calmness and happiness, though not perceived stress itself), and also reduced feelings of “fullness,” a marker of appetite. The increase in SBP is of public health significance, as repetitive elevation of SBP through regular video‐gaming in the absence of physical activity could affect long‐term regulation of BP, potentially leading to hypertension (Phillips & Hughes, 2011). The changes in mood that we detected (alert, calm, happy, stress) were of biological significance, amounting to changes of 0.4 to 0.9 z‐scores relative to baseline data. Collectively, these results indicated that activating the stress response might be increasing glucose demand in the brain. These findings provide further evidence for the hypothesis that violent content in video games can elicit a stress response with both immediate and lasting effects on cardiovascular load, consistent with two previous studies (Siervo et al., 2013; Siervo et al., 2018).

Despite these effects, the violent game did not affect physiological markers such as grip strength or memory recall, and did not result in a greater consumption of food after the video game intervention had ended. Overall, our results indicate that experimental activation of the stress response during sedentary activity does not increase functional outcomes of either of two tissues considered key to the “fight‐or‐flight” response, namely muscle tissue and brain. Instead, effects on the brain appeared to be restricted to specific markers of the stress response, such as increased “alertness” and decreased “calmness” and “happiness,” rather than impacting cognitive function. Our study also does not support the cerebral supply chain model, as there was no indication that muscles received fuel at a cost to the brain. However, it is also possible that our violent video game stimulus was too weak to generate the expected responses.

Interestingly, following the ad libitum snack period a change over time in fullness was observed between the two groups, with the violent game group experiencing a much higher rate of increase in fullness, despite not consuming more food. This may indicate lasting effects of greater cerebral glucose uptake following the violent game intervention. In other words, the brain's ability to “pull” glucose from the bloodstream may have led initially to reduced sensations of fullness in the violent game group, followed by a stronger satiety response following the consumption of food. Nonetheless, these differences did not drive greater food consumption to replenish the glucose consumed in association with the tress response.

The stress response may have positive as well as negative effects on mood, for example increasing excitement, and our findings for alertness and calmness merit consideration through that lens. A previous study found that playing a violent video game did not increase aggressive behavior relative to playing a nonviolent game, and that those habituated to playing violent games felt less hostile and depressed after the violent stimulus (Ferguson & Rueda, 2010). However, a desire for excitement has also been proposed to predispose some individuals towards violence (Howard, 2011), and many video games use violent content to generate thrill‐seeking experience. The stress response and the reward system are complex phenomena that are closely associated, and this link underlies the well‐established stimulating effect of stress on appetite (Adam & Epel, 2007; Schellekens et al., 2012).

Links between stress and appetite may involve systemic effects on the neuroendocrine system, mediated by effects on leptin, ghrelin, insulin, neuropeptide Y and gastrointestinal hormones (Michels, 2019). Under chronic stress conditions, the hypothalamic–pituitary–adrenal axis (HPA) is activated, resulting in corticosteroid release (Sharma et al., 2006; Sunram‐Lea et al., 2012; Torres & Nowson, 2007). High circulating cortisol levels can inhibit the reward system, resulting in an increase in energy intake, which in turn acts as a negative feedback mechanism on the HPA axis (Dallman et al., 2003; Gibson, 2012). The pivotal role of cortisol in determining energy intake is illustrated by the fact that adrenalectomy is accompanied by a reduction in food consumption whereas glucocorticoid administration results in an increase in food intake (Sinha & Jastreboff, 2013). Stress can also have an impact on hormones that stimulate or suppress appetite, namely ghrelin and leptin respectively. For example, stressful conditions have been shown to raise ghrelin and inhibit leptin signaling (Schellekens et al., 2012; Tomiyama et al., 2012). We were unable to measure hormones in this study, hence this would be a valuable angle for future research.

A previous study reported a strong association between playing nonviolent video games (FIFA/football) and increased mental workload (Chaput et al., 2008; Chaput, Visby, et al., 2011). The study also showed increased circulating glucose concentration in response to the video game, however this was not accompanied by an increase in cortisol concentrations. This study therefore linked mental workload with glucose dynamics but did not consider the mediating role of activating the stress response.

In our study, the two video games differed not only in violent content, but also in other ways. For example, the violent game could also be considered an action game, and differed in the pace of action and the nature of competition. It has been proposed for example that the element of competition, rather than violence, in video games triggers aggressive behavior (Adachi & Willoughby, 2011). However, this has little implication for our study. Violent games are major sellers in the video game market, and an analysis of mature‐rated games of different genres found that all contained violence, with 83% containing strong violence (Thompson et al., 2006). The violent game in our study activated the stress response as intended, and we suggest that many other video games may have the same effect, even if not overtly presented as a violent game. However, we deliberately chose a game with violent content for this study, as from an evolutionary perspective, both social competition and pursuing animal prey may involve violence.

Our own study aimed to complement existing theoretical frameworks with a life history perspective. The most likely explanation for our findings is that the brain may itself demonstrate selective allocation of fuel across competing cognitive functions. The VAS scores indicate that some functions were enhanced in those playing the violent game, as shown by greater levels of alertness and alterations in markers of mood, though not of perceived stress itself. However, there did not appear to be a more generalized increase in fuel allocation to the brain, as indicated by the lack of difference on the memory recall test. This suggests that the flight‐or‐fight response may exert quite specific effects on brain function, and that it may only activate those functions that are important for immediate survival. In other words, there may be fuel redistribution within the brain, rather than greater allocation to this organ.

Previous studies support the hypothesis that manipulating the stress response or other components of psychological state may alter cerebral fuel distribution. For example, a study using functional magnetic resonance imaging (fMRI) demonstrated contrasting activating/deactivating effects in specific brain structures in young adult men playing a violent video game (Mathiak & Weber, 2006). These effects were large in magnitude and appeared to differ from pure arousal or cognitive demand effects. Using a different stimulus, fMRI studies of meditation likewise demonstrate altered cerebral blood flow, with increased blood flow to the frontal lobe, and decreased flow to other regions such as the parietal and occipital lobes (Newberg et al., 2003; Wang et al., 2011).

The 26 kcal difference in energy intake between the groups was substantially smaller than that we had hypothesized (>227 kcal) following our earlier study (Siervo et al., 2018). An important difference between the studies comprises the weight status of the participants. It is possible that the effect of activating the stress response on food intake is greater in overweight/obese individuals than in those of normal BMI. In all three trials we have conducted to date, the violent video game group has reported reduced feelings of “fullness” relative to the nonviolent video game and TV‐watching groups, but this only translated into greater food intake in the study of overweight men. Therefore, there may be an interaction between the effects of the stress response on appetite, depending on the nutritional status and metabolic sensitivity of the participants.

Our study had potential limitations. First, our intervention was relatively brief, and the impact on the stress response may have been relatively modest. Any deficit in cerebral glucose relating to the intervention may have been resolved through normal blood supply. It is possible that a longer exposure to stress, or a more stressful intervention, might have led to a greater impact on appetite. We wanted to study a behavior that is highly prevalent, and hence chose a period that might represent the behavior of a regular video game player. However, we acknowledge that many video game participants do indeed play for much longer time periods, and this could be targeted by future studies.

Second, the test meal protocol assumed that the opening of a food packet led to complete consumption of its contents, and we did not directly obtain weighed food intakes. Perception of the taste of food may itself have been influenced by the intervention, which could have affected the amount of food consumed. Future studies could address this by providing only a single food item of homogenous content.

Third, the sample size of 35 and 36 in the two groups limited the potential to detect more subtle effects. It would have been ideal to investigate hormones associated with appetite. By recruiting men who habitually played video games, we may also have studied a sample habituated to violent games, who may therefore have demonstrated a reduced activation of the stress response. This would reduce our ability to detect differences between groups, but it also makes our results generalizable to young men who play video games regularly.

Fourth, the failure of the blood pressure cuff during the initial phase of the trial reduced the sample size, which might make the blood pressure results less reliable. However, we collected repeat measurements which showed stable group‐differences through the intervention. Moreover, the magnitude of increase in SBP (+4 mm Hg) in the violent group at the end of the study was very similar to that of +4.7 mm Hg that we detected in a previous comparison of video game players, compared to television‐watching controls (Siervo et al., 2018). We therefore think it unlikely that these results are unreliable.

Strengths of the study include the repeated collection of diverse outcome data over a short time period, and the direct testing of appetite using a test‐meal.

## 5 CONCLUSION

Our findings add to growing evidence that playing video games, in particular those with violent content, activates the stress response, and that this has some effect on sensations of appetite, though not necessarily on immediate energy intake. They also add to our understanding of inter‐tissue competition for fuel. Whereas an intense exercise load results in the relative preservation of brain function at the expense of muscular function (Longman et al., 2017), we found no indication of such inter‐organ competition when the stress response was activated in the absence of an exercise load, which we suggest might be due to reorganization of energy allocation within the brain. The mechanisms whereby stress‐inducing video games affect appetite, and potentially food intake and weight gain, merit further investigation.

### AUTHOR CONTRIBUTIONS

**Jonathan C. K. Wells:** Conceptualization (equal); formal analysis (equal); methodology (equal); writing – original draft (equal); writing – review and editing (equal). **Panos Michael:** Conceptualization (equal); data curation (equal); formal analysis (equal); investigation (equal); methodology (equal); writing – original draft (equal). **Mary S. Fewtrell:** Conceptualization (equal); methodology (equal); writing – review and editing (equal). **Mario Siervo:** Conceptualization (equal); methodology (equal); writing – review and editing (equal). **Mario Cortina‐Borja:** Formal analysis (equal); methodology (equal); supervision (equal); writing – original draft (equal); writing – review and editing (equal).

## CONFLICT OF INTEREST

All authors declare no conflict of interest.

## Supporting information


Figure S1

Table S1

## Data Availability

The data that support the findings of this study are available on request from the corresponding author. The data are not publicly available due to privacy or ethical restrictions.
