# Supplementary material for: Fright but not fight‐or‐flight: Violent video games elevated stress markers, but did not impact muscle function, memory recall or food intake, in a randomized trial in healthy young men
Source: Am J Biol Anthropol. 2022 May 24;178(3):476–87. doi: 10.1002/ajpa.24559 (PMC9546372; doi:10.1002/ajpa.24559)
Supplement: Supplementary file 1 — Figure S1 [file AJPA-178-476-s001.doc]

**CONSORT 2010 Flow Diagram**

**Allocation**

**Analysis**

**Enrollment**

Assessed for eligibility (n= 127)

Excluded (n= 46)

♦  Not meeting inclusion criteria (n= 31)

♦  Declined to participate (n= 19 )

♦  Failed final screening study (n= 6)

Analysed (n= 35)
♦ Excluded from analysis (give reasons) (n=0)

Allocated to violent video game (n= 35)

♦ Received allocated intervention (n= 35)

♦ Did not receive allocated intervention (give reasons) (n= 0)

Allocated to non-violent video game (n= 36)

♦ Received allocated intervention (n= 36)

♦ Did not receive allocated intervention (give reasons) (n= 0)

Analysed (n= 36)
♦ Excluded from analysis (give reasons) (n= 0)

Randomized (n= 71)
